# Physiological and Transcriptomic Analyses Uncover the Reason for the Inhibition of Photosynthesis by Phosphate Deficiency in *Cucumis melo* L.

**DOI:** 10.3390/ijms232012073

**Published:** 2022-10-11

**Authors:** Pengli Li, Jing Yu, Ningxiao Feng, Jinyang Weng, Asad Rehman, Jinyang Huang, Song Tu, Qingliang Niu

**Affiliations:** Key Laboratory of Urban Agriculture (South), Ministry of Agriculture, School of Agriculture and Biology, Shanghai Jiao Tong University, Shanghai 200240, China

**Keywords:** Pi deficiency, water content, phospholipid, cell membrane, chloroplast ultrastructure, transcriptomic analysis, photosynthesis pathways

## Abstract

Phosphate (Pi) deficiency is a common phenomenon in agricultural production and limits plant growth. Recent work showed that long-term Pi deficiency caused the inhibition of photosynthesis and inefficient electron transport. However, the underlying mechanisms are still unknown. In this study, we used the physiological, histochemical, and transcriptomic methods to investigate the effect of low-Pi stress on photosynthetic gas exchange parameters, cell membrane lipid, chloroplast ultrastructure, and transcriptional regulation of key genes in melon seedlings. The results showed that Pi deficiency significantly downregulated the expression of aquaporin genes, induced an increase in ABA levels, and reduced the water content and free water content of melon leaves, which caused physiological drought in melon leaves. Therefore, gas exchange was disturbed. Pi deficiency also reduced the phospholipid contents in leaf cell membranes, caused the peroxidation of membrane lipids, and destroyed the ultrastructure of chloroplasts. The transcriptomic analysis showed that 822 differentially expressed genes (DEGs) were upregulated and 1254 downregulated by Pi deficiency in leaves. GO and KEGG enrichment analysis showed that DEGs significantly enriched in chloroplast thylakoid membrane composition (GO:0009535), photosynthesis-antenna proteins (map00196), and photosynthesis pathways (map00195) were downregulated by Pi deficiency. It indicated that Pi deficiency regulated photosynthesis-related genes at the transcriptional level, thereby affecting the histochemical properties and physiological functions, and consequently causing the reduced light assimilation ability and photosynthesis efficiency. It enriches the mechanism of photosynthesis inhibition by Pi deficiency.

## 1. Introduction

Nutrient availability is very important for plant growth and production. Phosphorus (P) is a finite natural resource and an essential plant macronutrient with a major impact on crop productivity and global food security. P is involved in photosynthesis, carbon metabolism, and energy transfer. However, it is one of the least available macronutrients in soil [1]. It is estimated that 30% of the world’s agricultural soils are P-deficient and need fertilizer addition to ensure yield and quality [2]. Plants have evolved a series of morphological, physiological, and biochemical responses to enhance Pi acquisition from the environment and/or to remobilize orthophosphate [3].

Plant growth is dependent on mineral nutrient acquisition and photosynthesis. P is the composition of chloroplast and affects the structure and function of photosynthetic organs [4]. Thus, photosynthesis is sensitive to P deficiency. It was reported that P deficiency significantly decreases the leaf net photosynthesis rate in Arabidopsis [5], common bean [6], barley [7], rice [8], soybean [9], sugar beet [10], and *Arachis hypogaea* L. [11]. Inhibition of photosynthesis caused by P deficiency was mainly due to a smaller ATP content, the inhibition in ATP synthase activity [7], the decrease in ribulose 1,5-bisphosphate (RuBP) pool size [12], photodamage extending of the photosystem [11], disordered functional homeostasis of phosphate metabolism [13], and low sink demand [14]. In addition, P deficiency inhibited the linear electron flow in barley [7] and cyclic electron transport around PSI in melon [15], and decreased the electron transport rate in sheepgrass [16]. Our previous research found that low-Pi stress inhibited ATP synthase activity and disturbed the proton and electron transport efficiency and types in melon [15]. Although great progress has been made, the mechanism of these physiological and photochemical phenomena at the transcriptional and molecular levels is still unknown.

Transcriptome sequencing technologies can detect the molecular mechanisms of plant responses to abiotic stress at the whole genome level [17,18]. Some studies were conducted on plant responses to P deficiency in rice [19], maize [20], soybean [21], and wheat [22] at the transcriptome level. The differentially expressed genes (DEGs) were mainly involved in P metabolism, the oxidation-reduction process, the carbohydrate metabolic process, signal transduction [20], and acid phosphatase (APase) activity [23,24]. However, these pieces of research were focused on P-utilization efficiency and low-Pi tolerance in the root. The molecular mechanism for regulating photosynthesis in response to low-Pi remains poorly understood, particularly in melon (*Cucumis melo* L.), which is cultivated worldwide and requires massive amounts of Pi.

Although many aspects of the photosynthetic process are nowadays substantially elucidated, several details, specific regulations, and even structural details about photosynthesis in plants are still little known [25]. In this study, we performed the physiological, histochemical, and comparative transcriptomic analyses of leaves from melon plants treated with 0.25 mM (P_0.25_, control (CK)) and 0.001 mM (P_0.001_, low phosphate (LP)) Pi over 14 days. The goals of this study were (i) to investigate the physiological and molecular regulatory mechanism of melon photosynthesis in response to Pi deficiency; and (ii) to provide useful information for further research regarding the selection of the candidate genes involved in photosynthesis and Pi-deficiency responses.

## 2. Results

### 2.1. The Photosynthetic Light–Response Curve under Low-Pi Stress

**Figure 1:**
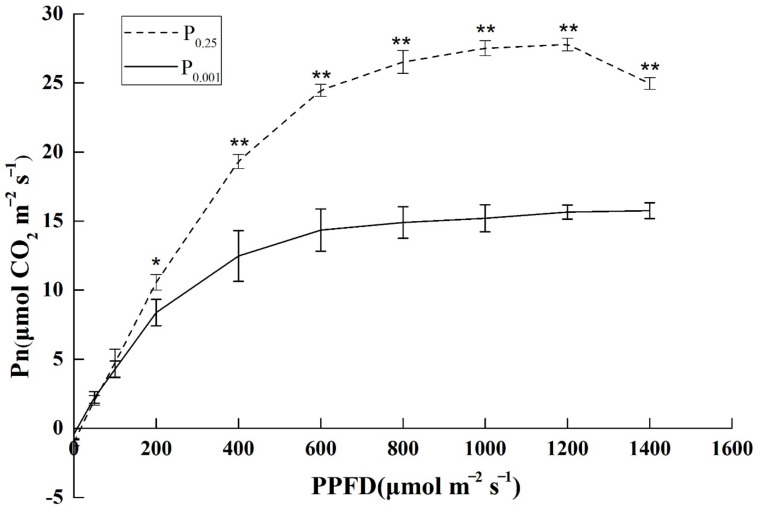
The seedling photosynthetic light–response curves under the different Pi treatments. Pn: net photosynthesis rate; PPFD: photosynthetic photon flux density. The error bars indicate the standard deviation (SD; *n* = 4). The asterisks indicate the significant differences according to Tukey’s test using a one-way ANOVA (* *p* < 0.05; ** *p* < 0.01).

Light–response curves showed a significantly lower net assimilation rate of Pi deficiency than CK (Figure 1). With the increase in photosynthetic photon flux density (PPFD), the net photosynthetic rate (Pn) of melon seedlings showed a nonlinear increasing trend. The ability to utilize weak light was significantly weaker under P_0.001_ stress than P_0.25_ from the slope of the curve. After PPFD reached 200 μmol m^−2^ s^−1^, Pn under P_0.001_ was significantly lower than CK. The maximum net photosynthetic rate (Pn_max_) of melon seedlings was reached at 600 μmol m^−2^ s^−1^ under P_0.001_. However, the Pn_max_ was reached at 800 μmol m^−2^ s^−1^ under P_0.25_. The saturated light intensity and Pn were reduced by Pi deficiency. It indicated that Pi deficiency significantly inhibited the light assimilation ability and photosynthesis efficiency of melons.

### 2.2. Leaf Water Content and Gas Exchange Parameters under Low-Pi Stress

**Figure 2:**
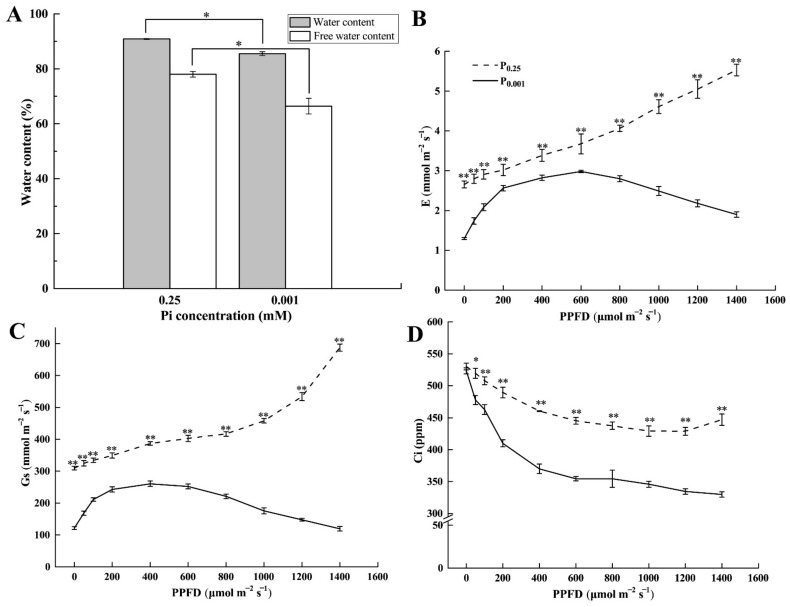
Leaf water content, free water content (**A**); E: transpiration rate (**B**); Gs: stomatal conductance (**C**); and Ci: intercellular CO_2_ concentrations (**D**) under the different Pi treatments. The error bars indicate SD (*n* = 4). The asterisks indicate the significant differences according to Tukey’s test using one-way ANOVA (* *p* < 0.05; ** *p* < 0.01).

To explore the reason for the photosynthesis inhibition under Pi deficiency conditions, we investigated the leaf water content under low-Pi stress. The water and free water content of leaves under P_0.001_ were significantly lower than the control by 5.89% and 14.88%, respectively (Figure 2A). Meanwhile, leaf transpiration rate (E) (Figure 2B), stomatal conductance (Gs) (Figure 2C), and intercellular CO_2_ concentrations (Ci) (Figure 2D) under P_0.001_ were significantly lower than CK. When PPFD was less than 600 μmol m^−2^ s^−1^, E increased with the increase in PPFD; after PPFD reached 600 μmol m^−2^ s^−1^, E gradually decreased with the increase in PPFD under P_0.001_. Gs showed the same trend with E under P_0.001_. However, the E and Gs increased over the PPFD under CK conditions. Ci decreased with the increase in PPFD. These results suggested that low-Pi stress induced the physiological drought in melon leaves and further caused E and Gs to decrease, which disturbed the gas exchanges.

### 2.3. The Endogenous Hormone Contents under Low-Pi Stress

**Figure 3:**
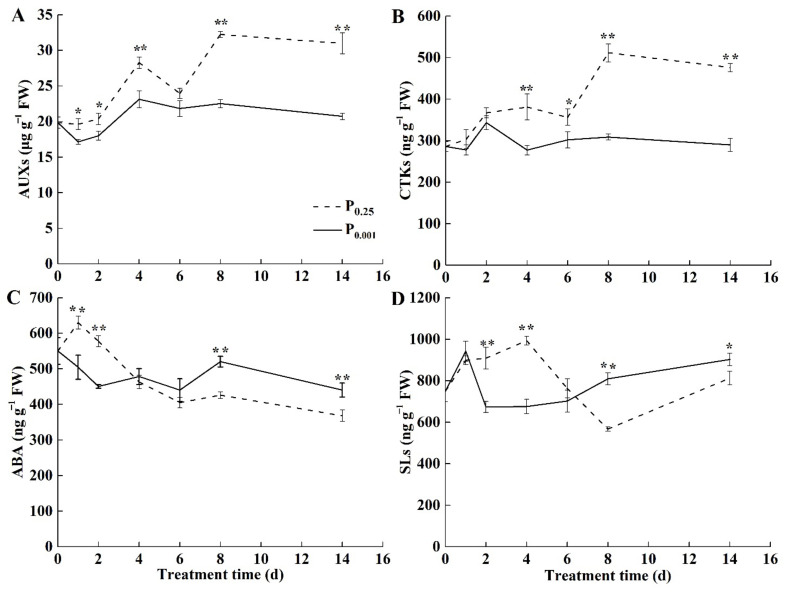
Changes in the endogenous hormone contents (**A**) AUX, (**B**) CTK, (**C**) ABA, and (**D**) SL in melon leaves. The error bars indicate SD (*n* = 4). The asterisks indicate the significant differences according to Tukey’s test using a one-way ANOVA (* *p* < 0.05; ** *p* < 0.01).

To explore the role of endogenous hormones in the photosynthesis inhibition under Pi deficiency, the contents of auxin (AUX), cytokinin (CTK), abscisic acid (ABA), and strigolactone (SL) in melon leaves were analyzed. Low-Pi stress significantly reduced AUX contents in melon leaves (Figure 3A). CTK contents in the leaves under low-Pi stress were significantly lower than CK after 4 days of treatment (Figure 3B). The decrease in the contents of AUX and CTK did not favor the growth and cell division of the leaves and caused the smaller sink demand. ABA contents under low-Pi stress were gradually higher than CK after 4 days of stress and significantly higher than CK on the 8th and 14th days (Figure 3C). SL contents in the leaves were 1.43-fold and 1.11-fold higher than CK on the 8th and 14th days under low-Pi stress (Figure 3D). It inhibited leaf growth and lateral branch sprouting and reduced the sink demand for leaf growth.

### 2.4. Cell Membrane Lipids of the Melon Leaf under Low-Pi Stress

**Figure 4:**
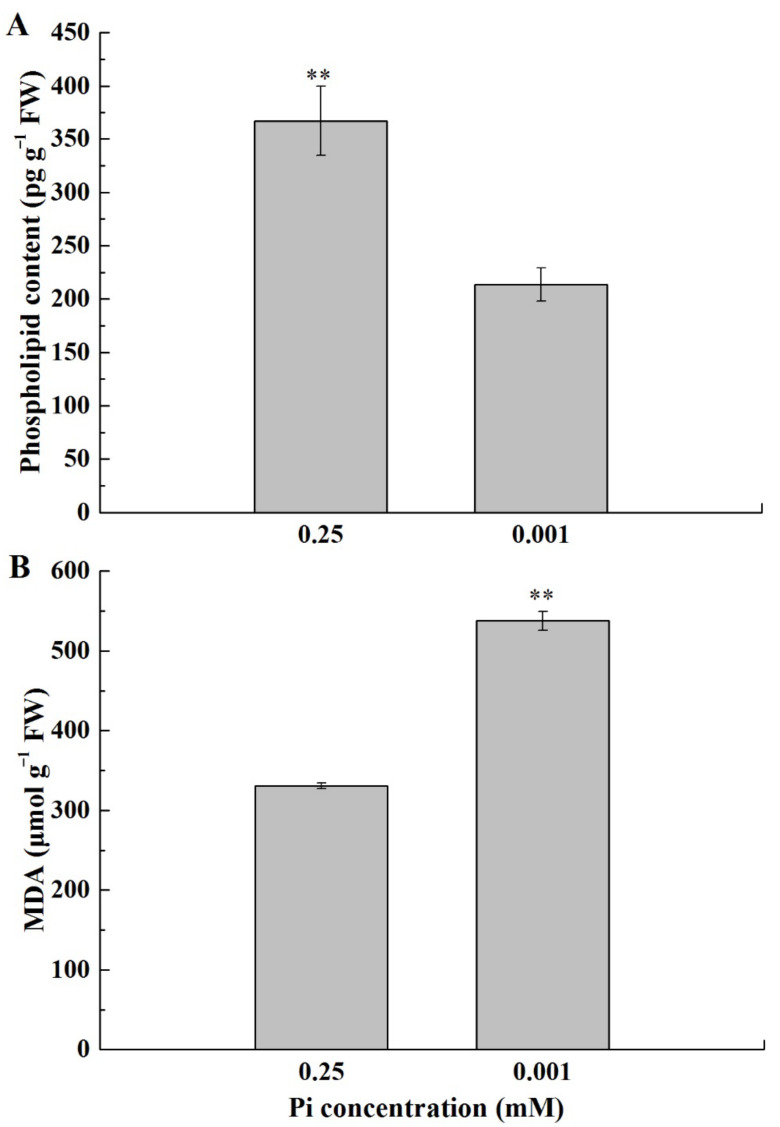
Phospholipid contents (**A**) and MDA contents (**B**) in the first true leaf under the different Pi treatments. The error bars indicate SD (*n* = 4). The asterisks indicate the significant differences according to Tukey’s test using a one-way ANOVA (** *p* < 0.01).

To explore the effect of low-Pi stress on leaf cell membrane phospholipid, phospholipid contents were measured (Figure 4A). The phospholipid content under P_0.001_ was significantly lower than CK. Low-Pi stress limited the phospholipid reserves and was harmful to maintaining the membrane integrity. In addition, the malondialdehyde (MDA) content under P_0.001_ was much higher than CK (Figure 4B), suggesting that low-Pi stress caused cell membrane lipid peroxidation. 

### 2.5. The Morphology of Mesophyll Cells and Chloroplasts under Low-Pi Stress

**Figure 5:**
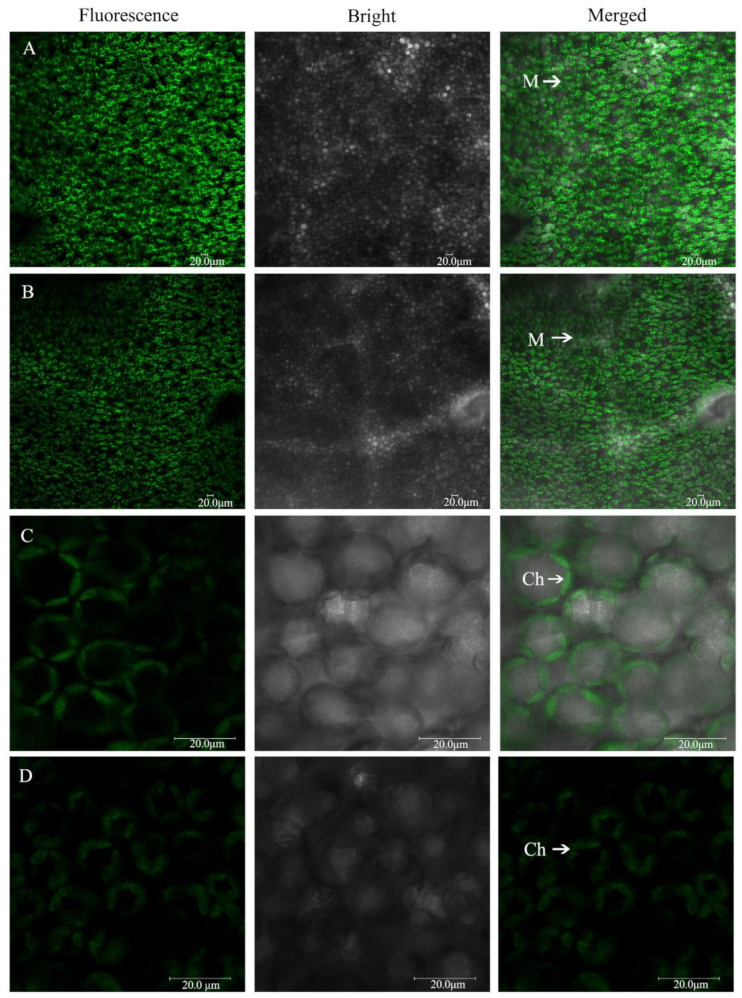
The morphology of the mesophyll cells (**A**): P_0.25_; (**B**): P_0.001_ and chloroplasts; (**C**): P_0.25_; (**D**): P_0.001_ imaged by confocal laser-scanning microscopy under the different Pi treatments. M: mesophyll cell; Ch: chloroplast. Scale bars, 20 μm.

The intact mesophyll cells and chloroplasts were visualized and imaged using confocal laser-scanning microscopy under chloroplast autofluorescence (Figure 5). The mesophyll cells were large and closely arranged under P_0.25_ (Figure 5A). However, mesophyll cells were small and loosely arranged under P_0.001_ (Figure 5B). The chloroplasts were spindle-shaped and their spread fitted to the shape of the cell wall and was in close contact with the adjacent chloroplasts under P_0.25_ (Figure 5C). The chloroplasts were shorter, expanded oval-shaped, and occupied a large amount of space in the mesophyll cells under P_0.001_ (Figure 5D). This finding suggested that Pi deficiency played a negative role in the development of chloroplasts and mesophyll cells.

### 2.6. Chloroplast Ultrastructure under Low-Pi Stress

**Figure 6:**
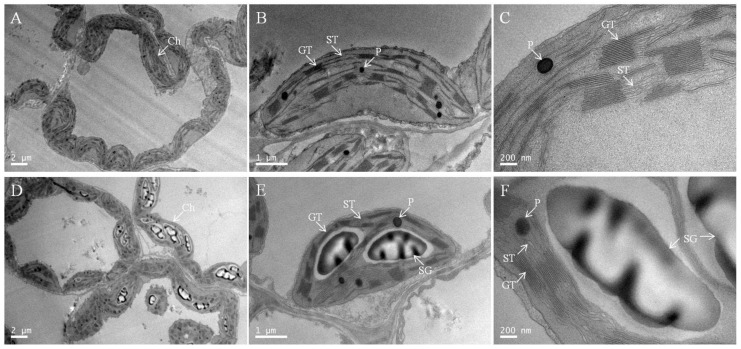
Representative electron micrographs of chloroplasts taken from the intact leaves of P_0.25_ (**A**–**C**) and P_0.001_ (**D**–**F**). CW, cell wall; GT, grana thylakoid; ST, stroma thylakoid; SG, starch granule; P, plastoglobuli. The scales for the mesophyll cells (**A**,**D**), the chloroplast (**B**,**E**), and the enlarged parts (**C**,**F**) are 2 μm, 1 μm, and 200 nm, respectively.

To elucidate the further effect of low-Pi stress on chloroplasts, we examined the chloroplast ultrastructure (Figure 6 and Table 1). Chloroplasts under P_0.25_ had well-developed inner membrane systems consisting of numerous grana, relatively long stromal thylakoids, abundant chloroplast matrices, relatively fewer osmiophilic plastoglobules, and no starch granules (Figure 6A–C and Table 1). The chloroplasts under P_0.001_ had comparatively less developed membrane systems with shorter, thinner, and fewer grana and stroma lamellae, less chloroplast matrix, more osmiophilic plastoglobules, and starch grains occupying large spaces of the chloroplasts (Figure 6D–F and Table 1). A magnified view showed that the lamellae were disintegrated, the grana were dissolved, and the thylakoids were swollen and disorganized as a result of the accumulation of oversized starch grains under low-Pi stress (Figure 6F). Pi deficiency damaged the chloroplast ultrastructure.

### 2.7. Transcriptome Analysis of DEGs, GO and KEGG Enrichment in the Melon Leaf 

**Figure 7:**
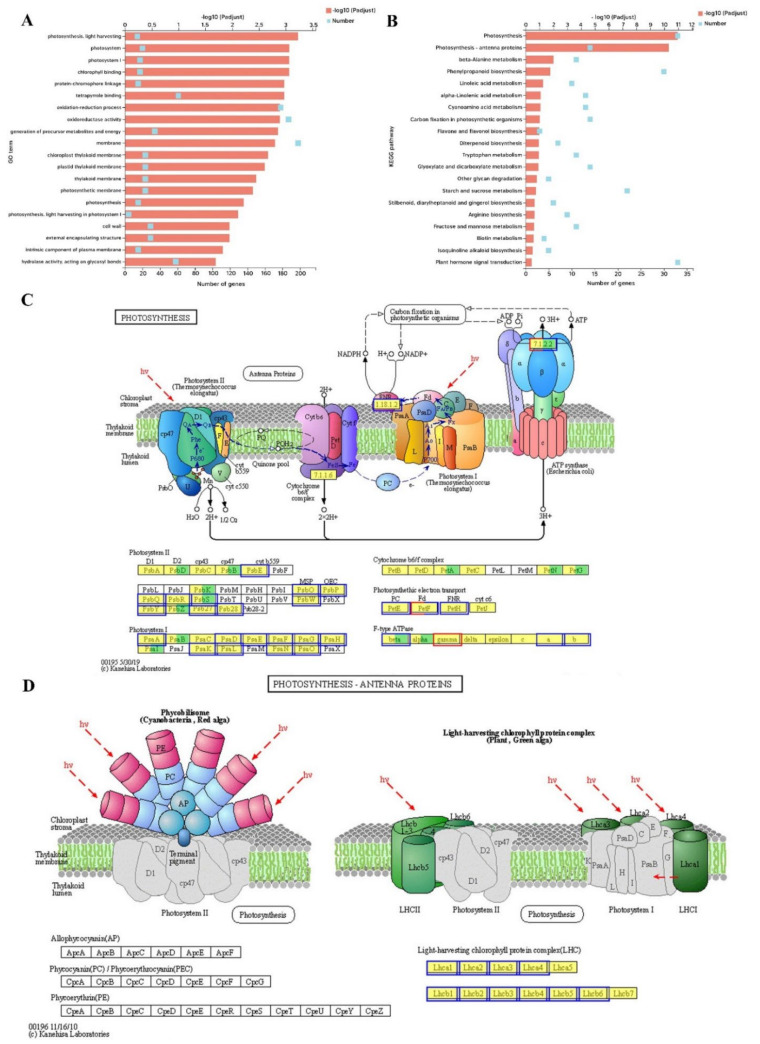
GO and KEGG functional enrichment analysis under low-Pi stress. (**A**) Top 20 GO terms of DEG enrichment under low-Pi stress. (**B**) Top 20 KEGG pathways of DEG enrichment under LP stress. (**C**) Schematic diagram of photosynthesis KEGG pathway. The yellow and green backgrounds indicate the known and new genes, respectively. The red and blue borders indicate the up- and downregulated genes, respectively. (**D**) Schematic diagram of photosynthesis-antenna proteins KEGG pathway. The yellow backgrounds indicate the known genes. The blue borders indicate the downregulated genes.

To analyze the further reason for the impeded photosynthesis, transcriptome-level differences were explored between low-Pi and CK leaves. In total, 822 DEGs were upregulated and 1254 downregulated at |log_2_FC| ≥ 1 and a false discovery rate (FDR) < 0.05 as thresholds (Figure S1 and Table S2). GO functions were mainly enriched in the cellular component including photosystem (20 DEGs: 20 down), membrane (198 DEGs: 74 up and 124 down), chloroplast thylakoid membrane (23 DEGs: 23 down), cell wall (29 DEGs: four up and 25 down), and intrinsic component of the plasma membrane (15 DEGs: six up and nine down). GO functions in the biological process included photosynthesis light-harvesting (14 DEGs: 14 down), protein-chromophore linkage (15 DEGs: 15 down), oxidation-reduction process (178 DEGs: 76 up and 102 down), and photosynthesis (15 DEGs: 15 down). GO functions on the molecular function including chlorophyll-binding (17 DEGs: 17 down) and oxidoreductase activity (187 DEGs: 82 up and 105 down) (Figure 7A). The KEGG enrichment pathways of DEGs were significantly enriched in photosynthesis (33 DEGs) and photosynthesis-antenna proteins (14 DEGs) (Figure 7B). The DEGs on the photosynthesis pathway were mainly focused on PsbE, PsbO, PsbP, PsbQ, PsbR, PsbW, PsbY, Psb27, Psb28 of photosystem II; PsaA, PsaB, PsaD, PsaF, PsaG, PsaH, PsaK, PsaL, PsaN, PsaO of photosystem I; PetE of PC; PetF of Fd; PetH of FNR; subunit beta, ATPFOA, and ATPFOB of F-type ATPase (Figure 7C). The DEGs enriched in the photosynthesis-antenna proteins pathway were the Lhca1-4 of photosystem I and Lhcb1-6 of photosystem II (Figure 7D). These indicated that Pi deficiency strongly affected various regulatory pathways involved in the photosynthesis of the melon leaf.

### 2.8. Transcriptional Regulation of Low-Pi Stress to Genes on Photosynthesis

**Figure 8:**
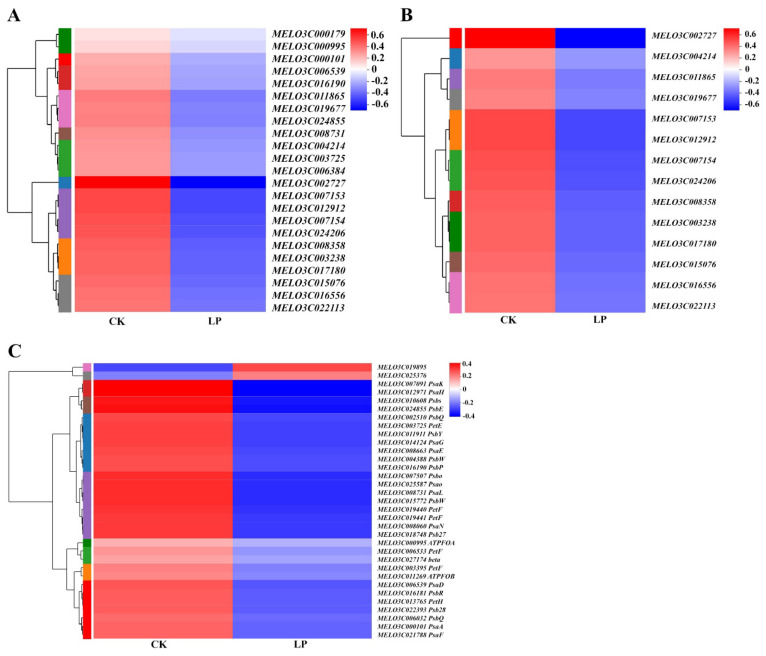
The hierarchical cluster and heatmap of DEGs enriched in the chloroplast thylakoid membrane (**A**), photosynthesis light-harvesting (**B**), and photosynthesis pathway (**C**) based on the log_10_ (fold change) values. Genes with a fold change ≥ 2 and an FDR < 0.05 were considered to be DEGs. Blue and red bands were used to represent low and high expression levels, respectively.

The clustering heatmap of DEGs based on the log_10_ (fold change) values showed that the DEGs on chloroplast thylakoid membrane and photosynthesis light harvesting were downregulated by low-Pi stress (Figure 8A,B). The DEGs enriched in photosynthesis were downregulated by low-Pi stress, with the exception of *MELO3C19895* and *MELO3C025376* (Figure 8C). The downregulated genes caused the functional disturbance of thylakoid membrane proteins, the light-harvesting complex, and photosynthetic chain proteins. Low-Pi stress played a negative role in the transcriptional regulation of genes on photosynthesis. It provided evidence of photosynthesis inhibition by low-Pi stress at the transcriptome level.

### 2.9. LP Induces the Downregulation of Aquaporin Genes at the Transcriptional Level

**Figure 9:**
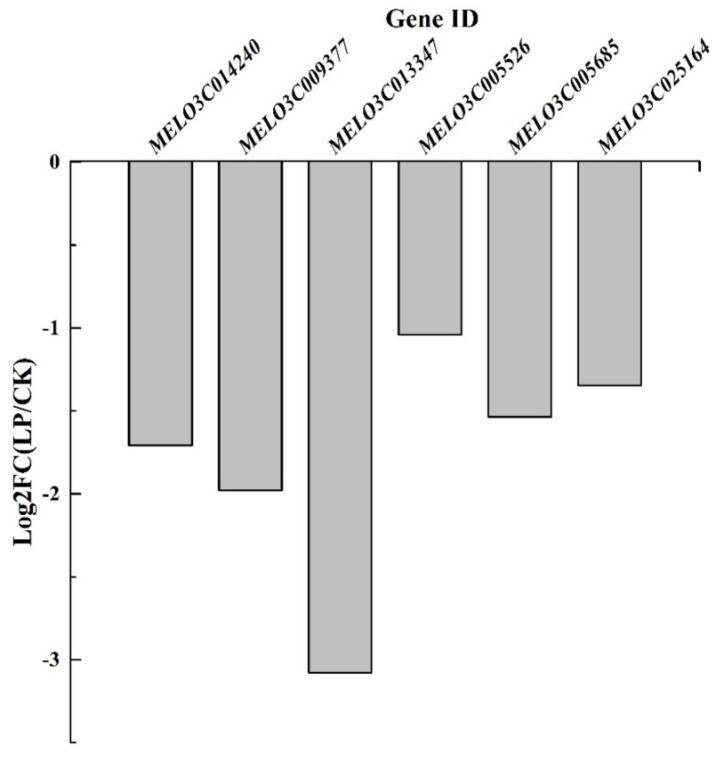
The log_2_ (fold change) of the aquaporin genes at the transcriptional level.

The aquaporin genes, *MELO3C014240*, *MELO3C009377*, *MELO3C013347*, *MELO3C005526*, *MELO3C005685*, and *MELO3C025164* were downregulated by P_0.001_ on the 14^th^ day of LP treatment (Figure 9). Low-Pi stress caused transcriptional downregulation of aquaporin genes in melon leaves. It was detrimental to H_2_O and CO_2_ exchange among mesophyll cells and chloroplasts.

### 2.10. Validation of RNA-Seq Results by Quantitative Real-Time PCR

**Figure 10:**
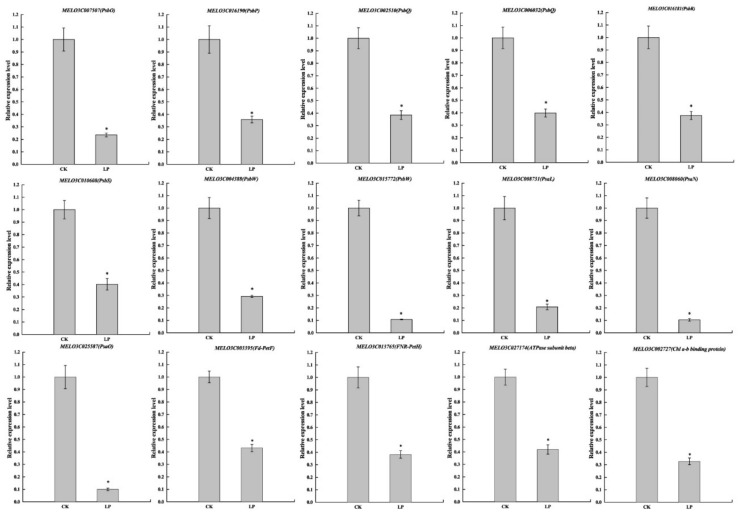
qRT-PCR validation of the selected DEGs. The error bars indicate SD (*n* = 6). The asterisks indicate the significant differences according to Tukey’s test with one-way ANOVA (* *p* < 0.05).

The qRT-PCR analysis was employed to validate the expression profiles of DEGs with 14 genes on the photosynthesis pathway and one gene on light harvesting (Figure 10). The relative expression level of all 15 genes was significantly lower than CK, which was characterized by a similar trend of transcript level. In conclusion, although the expression values were, in most cases, different from RNA-Seq in qRT-PCR, the obtained data confirmed the reliability of the results generated by RNA-Seq analysis.

## 3. Discussion

P participates in the photosynthesis in plants. The previous study showed that Pi deficiency disturbed the electron transport pathways and inhibited photosynthesis [15]. The physiological and molecular mechanisms were explored in this article. New insights revealed in the present study are discussed below.

### 3.1. Pi Deficiency Caused Physiological Drought in the Melon Leaf

Many experimental studies show that P deficiency results in decreased photosynthesis [4,7,15]. Our data support this finding (Figure 1). However, no experiments have been reported so far that concern the physiological drought caused by Pi deficiency in the melon. The water content in leaves was significantly lower under Pi deficiency than CK (Figure 2A). The levels of ABA, a phytohormone that mediates drought responses, increased upon Pi deficiency (Figure 3C) [26]. Consequently, the reduction of E, Gs, and Ci in the leaf occurred under Pi deficiency (Figure 2). These morphological features are typically found in drought-adapted species [27] and were also found in beech under Pi deficiency [28]. 

Meanwhile, Pi deficiency induced the downregulation of aquaporin genes at the transcriptional level (Figure 9). It is detrimental to the expression of aquaporin proteins. It further inhibited the transport of water and exchange of gases in the leaves [29]. Aquaporins play a role in altering leaf stomata movement. The reduced amount of NtAQP1 contained in the tobacco (*Nicotiana tabacum*) plasma membrane and inner chloroplast membranes caused a 20% change in CO_2_ conductance within leaves [30]. It showed that aquaporins in the chloroplast membrane regulated leaf internal CO_2_ transport. NtAQP1 overexpression heightened membrane permeability for CO_2_ and water and increased leaf growth [31]. Hordeum vulgare HvPIP2;1, HvPIP2;2, HvPIP2;3 and HvPIP2;5 facilitated CO_2_ transport across the oocyte cell membrane [29]. It was concluded that Pi deficiency caused physiological drought in the melon leaf, induced the drought stress-like responses and further decreased photosynthesis.

### 3.2. Pi Deficiency Disrupts the Morphology and Ultrastructure of the Chloroplasts

Complete configuration and structure in chloroplasts are required for ensuring normal leaf photosynthesis [32]. A higher photosynthetic rate benefits from more robust chloroplasts and thylakoid membranes [33]. Pi deficiency resulted in shorter chloroplasts, fewer grana stacks, broken stroma lamellae and the accumulation of starch grains in the stroma (Figure 6D–F). These results are largely consistent with earlier observations that low-Pi stress severely damaged the soybeans’ chloroplasts [34], reduced the number of thylakoid grana stackings in sheepgrass [16], and larger starch grain in aquatic fern *Azolla caroliniana* [35]. Low-Pi stress severely reduces the light interception and assimilation capacity of plants.

As an important component of membrane phospholipids, a decrease in P content in the leaf by Pi deficiency limited the phospholipid reserves (Figure 4A). Cellular membrane phospholipids represent one of the major storehouses that carry approximately 15–30% of total cellular organic P [36]. To utilize this P, Pi-starved plants initiate mechanisms that help in the extraction of Pi from P-lipids with minimal or no damage to the membrane functions. Widespread occurrence in diverse life forms suggests membrane lipid remodeling as a conserved mechanism crucial for survival under Pi deficiency [37]. During membrane lipid remodeling, P-free lipids, especially galacto- and sulpholipids, play a vital role in replacing membrane phospholipids and allowing hydrolysis of P-lipids to release Pi for essential cellular processes [38]. However, these replacements are still unable to meet the photosynthetic requirements of the melon from the chloroplast ultrastructure (Figure 5D–F). Pi deficiency also caused cell membrane lipid peroxidation (Figure 4B). The reduction in lipid biosynthesis and degradation of lipids caused the structure of the chloroplast membrane systems to be greatly disrupted and hence suppression of photosystem activity and photosynthesis in Pi-deficient plants [9,15].

### 3.3. Plastoglobuli Serve as Dynamic Lipid Reservoirs for Thylakoid Membranes under Pi Deficiency

Plants have developed chloroplast plasticity to help optimize the balance between photosynthesis and self-protection under changing environments [39]. Pi deficiency significantly increased the abundance of plastoglobuli (Figure 6 and Table 1), which is consistent with more abundant plastoglobuli under unfavorable conditions [40,41]. Plastoglobuli are physically attached to the thylakoid membrane, which can regarded as thylakoid-associated lipid microcompartments filled with lipophilic metabolites and enzymes [40]. Pi deficiency resulted in the reduction of lipid biosynthesis and acceleration of lipid degradation (Figure 4). Melon chloroplasts developed more plastoglobuli to maintain the normal lipid: protein ratio, which is crucial for thylakoid membrane functions for light harvesting [42]. If the amount of lipids in the thylakoid membranes is too low, the plastoglobuli cause the lipids to increase the lipid: protein ratio. In contrast, plastoglobuli take up excess lipids. In this way, a dynamic exchange of lipids between thylakoid membranes and plastoglobuli can adjust the lipid: protein ratio. This mechanism would allow fast adjustments to stress.

### 3.4. The Regulation of Pi Deficiency to Genes on the Photosynthesis at the Transcriptional Level 

Photosynthesis occurs in chloroplasts containing 3000–4000 different proteins. A small number of them are encoded by the plastid genome while the majority are encoded in the nucleus [43]. Expression of these genes, therefore, requires high coordination between the nucleus and chloroplasts. This is achieved by a bilateral information exchange including nucleus-to-plastid and plastid-to-nucleus signals [43]. The repressed photosynthetic capacity was not only caused by disturbed morphological and anatomical structure but also by the gene expression difference at the transcriptional level. Pi deficiency downregulated the expression of thylakoid membrane-related genes and photosynthetic electron transport chain genes of photosystem located in thylakoid membranes at the transcript level (Figure 8A,B). This was consistent with the fewer grana stacks and broken stroma lamellae (Figure 6F). It was reported that the redox state of plastoquinone controls the rate of transcription of genes encoding reaction-center apoproteins of PS I and PS II [43,44]. Our previous study found that Pi deficiency perturbed photosynthetic electron flow and the redox state of the plastoquinone pool [15]. In this study, the transcription of genes encoding the reaction center of PS II and PS I was downregulated under Pi deficiency (Figure 8D and Figure 9C). We speculate that the redox state of plastoquinone controls the transcription of genes encoding reaction-center apoproteins of PS I and PS II in melons. The light-harvesting complex (LHC) is involved in both light harvesting and photoprotection [45]. The downregulation of light-harvesting protein complex-related genes under low-Pi conditions (Figure 9B) is consistent with a reduced light-utilization capacity and alleviates light damage [15]. Thus, the downregulated expression of photosynthesis-related genes under low-Pi stress reduces light interception capacity and photosynthesis and protects the photosynthetic organ in melons.

## 4. Materials and Methods

### 4.1. Plant Material and Phosphate Treatment 

The melon cultivar ‘Lvtianshi’ was selected because it is widely planted south of the Yangtze River in China, where Pi deficiency is common. Hydroponic experiments were conducted in the greenhouse of Shanghai Jiao Tong University. Uniform seedlings with fully expanded cotyledons were transplanted into 15 L shallow-mouth plastic trays (40 plants per tray) filled with 10 L half-strength modified Hoagland’s nutrient solution. The nutrient solution consisted of 3 mM KNO_3_, 2.5 mM Ca (NO_3_)_2_, 1.0 mM MgSO_4_·7H_2_O, 25 μM KCl, 12.5 μM H_3_BO_3_, 1 μM MnSO_4_, 1 μM ZnSO_4_, 0.25 μM CuSO_4_, 0.25 μM H_2_MoO_4_ and 13.4 μM FeSO_4_/Na_2_EDTA with pH of 6.4 ± 0.2. Based on the previous research and preliminary experiment, 0.001 mM Pi (P_0.001_, LP) was selected as the dose for Pi-deficiency treatment, whereas the control (CK) was 0.25 mM Pi (P_0.25_, CK) [46]. Low-Pi media were prepared by substituting K_2_SO_4_ for KH_2_PO_4_ such that the concentration of K in the media was 3 mM for all treatments. There were seven trays per biological replicate with three replicates per treatment. The nutrient solutions were renewed every three days (d), and the trays were also rearranged randomly. The plants were cultivated in a growth cabinet with 28 °C/18 °C day/night temperature, 14 h light/10 h dark, and relative humidity of 50–75%. The experiment was established as a completely randomized design. At 0 d, 1st d, 2nd d, 4th d, 6th d, 8th d and 14th d after treatment, samples were harvested and prepared for morphological, physiological, and biochemical assays, with at least three sub-samples per assay.

### 4.2. Measurement of Gas Exchange Parameters 

The gas exchange parameters were measured between 9:00 am and 11:00 am on the first fully expanded true leaf of 14th d with a portable photosynthetic measurement system (GFS-300, HeinzWalz, Effeltrich, Germany) under the condition of 25 °C, relative humidity 70%, a cuvette air flow rate of 750 mL min^−1^, and an ambient CO_2_ concentration. Light–response curves were measured across 10 light conditions (0, 50, 100, 200, 400, 600, 800, 1000, 1200, and 1400 μmol m^−2^ s^−1^ photosynthetic photon flux density). 

### 4.3. Measurement of Leaf Water Content

The leaf water content (WC) was calculated according to the following formula: WC = 100% × (FM—DM)/FM, where DM and FM denote dry weight and fresh weight, respectively [47].

The determination of free water content is as follows: Take three weighing bottles (three repetitions), number, and weigh them, respectively. Select the uniform plants and take the representative leaves with the same position, growth status, and leaf age. Use a punch with a diameter of 0.5 cm to drill the leaf discs avoiding the veins. Pack 50 pieces randomly into each bottle, cap immediately, and weigh the fresh weight (mF). Quickly add about 5 mL 65–75% sucrose solution to each bottle (carefully shake well and not overlap the discs), and weigh them (mB). Keep the bottles dark for 4–6 h and shake them frequently. Then, use the Abbe refractometer to measure the concentration of the sugar solution B2 (%) and the original sugar solution concentration B1 (%). Then, calculate the free water content of plant tissues according to the formula: β = 100% × mB (B1-B2)/(B2 × mF).

### 4.4. Measurement of the Endogenous Hormone Contents

Endogenous hormone contents were determined by enzyme-linked immunosorbent assay (ELISA) as described by Han et al. (2015) [48]. Fresh melon leaves (0.5 g) were ground into homogenate on ice using extraction buffer (80% methanol containing 1 mM di-tert-butyl-p-cresol BHT). The homogenate was transferred into a 10 mL centrifuge tube and incubated at 4 °C in a shaker overnight. The mortar was then rinsed with 3 mL of extract, transferred to a test tube, shaken, and then placed in a refrigerator at 4 °C overnight. The samples were then centrifuged at 3500 rpm for 8 min and the supernatant was filtrated with a C-18 solid phase extraction column. The resulting sample was transferred to a small 25 mL beaker and the methanol in the extract was removed by vacuum freeze drying. The final volume was then adjusted to 1 mL with dilution buffer. All ELISA kits were provided by Shanghai Enzyme-linked Biotechnology Co., Ltd. (Shanghai, China).

### 4.5. Measurement of Phospholipid Content and Lipid Peroxidation (Malondialdehyde Content)

The plant phospholipids were determined by using the double-antibody sandwich method. The plant phospholipid purified was used to capture the antibody and coat the microplate to make a solid-phase antibody. Add the melon phospholipid to the coated microwell, and it was combined with the HRP-labeled detection antibody to form an antibody–antigen–enzyme-labeled antibody complex. Add the substrate TMB to color after thoroughly washing the microplate. TMB was converted to blue under the catalysis of the HRP enzyme and then to yellow under acid conditions. The shade of color was positively correlated with the phospholipid content in the sample. The absorbance (OD) was measured at a wavelength of 450 nm, and plant phospholipid content was calculated according to the standard curve. All kits were provided by Shanghai Enzyme-linked Biotechnology Co., Ltd. (Shanghai, China).

Malondialdehyde (MDA) content was determined according to Siripornadulsil et al. (2002) with modifications [49]. Briefly, leaf samples (0.3 g) were homogenized with 5 mL of 5% (*w*/*v*) trichloroacetic acid (TCA) followed by centrifugation at 3000× *g* and 4 °C for 15 min. For the assay, 0.2 mL of the supernatant was mixed with 0.5 mL of 0.5% thiobarbituric acid (TBA) solution and boiled for 10 min. The reaction was stopped by placing the mixture on ice. The mixture was then centrifuged at 3000× *g* and 4 °C for 15 min. The absorbance of the resulting supernatant was recorded and corrected for nonspecific turbidity at 532 nm by subtracting it from the absorbance at 450 and 600 nm.

### 4.6. Confocal Laser-Scanning Microscopy 

Confocal analysis was performed as described previously [50]. The chlorophyll autofluorescence was observed with an excitation wavelength of 480 nm using a confocal microscope (Leica Microsystems, Wetzlar, Germany). 

### 4.7. Chloroplast Ultrastructure

Transmission electron microscopy (TEM) was performed as described by Zhao et al. with a minor modification [51]. The first true leaves were cut into small sections (1–2 mm in length) and then fixed in 2.5% glutaraldehyde under 4 °C overnight. The tissue samples were washed 3× in 0.1 M phosphate buffer and fixed in 2% osmic acid (OsO_4_) at 4 °C for 2 h. The tissues were washed three times with 0.1 M phosphate buffer and then dehydrated with an ethanol series of 50% *v*/*v*, 70% *v*/*v*, and 90% *v*/*v* for 15 min per ethanol concentration. The tissue samples were infiltrated with mixtures of 90% ethanol and 90% acetone (1:1), 90% acetone, and 100% acetone, acetone and resin (1:1, 1:2), and 100% resin for 12 h. The tissue samples were embedded and polymerized at 60 °C for 48 h. Thin sections (50–70 nm) were prepared with a cryo-ultramicrotome (UC6-FC6, Leica Microsystems, Wetzlar, Germany) and double-stained with uranyl acetate-lead citrate. The tissue samples were examined under a transmission electron microscope (Tecnai G2 Spirit BioTWIN, Thermo Fisher Scientific, Waltham, MA, USA).

### 4.8. RNA Extraction, cDNA Library Construction and Sequencing

The first true leaf on the 14th day of treatments was selected to be used for RNA-seq analysis. Each treatment was replicated three times. Total RNA was extracted from leaves according to the operation method of RNAprep Pure Plant Plus Kit (Tiangen Biotech, Beijing, China). The high-quality RNA samples were used to construct cDNA library. Attached-oligo (dT) magnetic beads were used to purify mRNA from the total RNA before fragmentation of the mRNA. Subsequently, mRNA was broken into short fragments by adding fragmentation buffer. One-strand cDNA was synthesized with six-base random primers using mRNA as the template, and then two-strand cDNA was synthesized by adding buffer, dNTPs and DNA polymerase I. Subsequently, double-strand cDNA was purified using AMPure XP beads. The cDNA library was performed on the Illumina NovaSeq 6000 (2 × 150 bp read length) for transcriptome sequencing (Majorbio, Shanghai, China). The clean reads were selected by removing low-quality sequences, reads with more than 5% N bases, and adapter sequences. The clean reads were mapped to *Cucumis melo* L. reference genome (http://cucurbitgenomics.org/organism/18, accessed on 1 December 2021) by HISAT2 software (http://ccb.jhu.edu/software/hisat2/index.shtml, accessed on 1 December 2021) with mapping rate higher than 65% (Total Mapped Reads) [52] and assembled using Cufflinks (http://cole-trapnelllab.github.io/cufflinks/, accessed on 1 December 2021) [53]. The RNA sequence data set are available in the repository of NCBI Sequence Read Archive (SRA) under the accession BioProject number PRJNA875218.

### 4.9. Differential Gene Expression Analysis, GO (Gene Ontology) Term, and KEGG Pathway Analysis

Identification of differentially expressed genes (DEGs) was carried out using DESeq2 software. The DEGs with |log_2_FC| ≥ 1 and a false discovery rate (FDR) < 0.05 for either of the sample in each pair-wise comparison were considered to be significantly differentially expressed [54]. FPKM (expected number of fragments per kilobase of transcript sequence per million base pairs sequenced) was used to compare different expression genes (DEGs) in two samples [55].

GO enrichment analysis of genes in the gene group was performed using the software Goatools (https://github.com/tanghaibao/GOatools, accessed on 17 January 2022) to obtain which GO functions were predominantly present in the gene group [56]. GO function was considered significantly enriched with Fisher’s exact test when the *p*-value (P adjust) was <0.05. The KEGG pathway enrichment analysis was performed on the genes/transcripts in the gene set using the R script in Majorbio Cloud Platform [57]. When the *p*-value (P adjust) was <0.05, the KEGG pathway function was considered to be significantly enriched.

The cluster analysis of the GEGs based on the log_10_ (fold change) values was generated using RSEMsoftware package with hierarchical clustering [55].

### 4.10. Verification of DEGs by qRT-PCR

To verify the accuracy and repeatability of the RNA-seq data, 15 DEGs were selected for qRT-PCR validation. The total RNA was extracted from the first true leaf on the 14th d after Pi deficiency treatment by using RNAprep Pure Plant Plus Kit (Tiangen Biotech, Beijing, China), and cDNA was synthesized using PrimeScript^TM^ RT Master Mix. Quantitative RT-PCR was performed using an SYBR^®^ Premix Ex TaqTM Kit (Tiangen Biotech, Beijing, China) with a Roche LightCycler 96 real-time PCR machine (Roche, Basel, Switzerland) with six replicates. The relative expression was calculated using the 2^−∆∆Ct^ method [58]. Actin was used as an internal control [59]. The primers used for qRT-PCR were listed in Supplementary Table S1.

### 4.11. Statistical Analyses

Statistical analysis was performed in SPSS Statistics 22.0 (IBM, Chicago, IL, USA). The means were analyzed using one-way ANOVA with Tukey’s test (*p* < 0.05). All figures were drawn with OriginPro 2016 (OriginLab, Northampton, MA, USA).

## 5. Conclusions

Pi deficiency downregulated the expression of aquaporin genes and induced an increase in ABA levels in leaves, which caused physiological drought in the leaves. It further decreased E, Gs, and Ci under high light intensity and reduced photo assimilation capacity and photosynthesis rate. In addition, Pi deficiency reduced the phospholipid contents in leaf cell membranes, caused the peroxidation of membrane lipids, destroyed the ultrastructure of chloroplasts, and further inhibited photosynthesis. Transcriptional analysis showed that *Lhca1-4* of LHC I and *Lhcb1-6* of LHC II protein synthesis genes were downregulated by Pi deficiency at the transcriptome level, resulting in decreased light capture ability. Meanwhile, the genes on the photosynthesis electron transport chain were also downregulated by Pi deficiency, which perturbed proton and electron transport efficiency. The redox state of plastoquinone could control the transcription of genes encoding the reaction-center apoproteins of PS I and PS II in melons. This study provides new mechanisms of photosynthesis inhibition by Pi deficiency. Since P deficiency results in physiological drought, and decreases stomatal conductance and cellular CO_2_ concentrations, we suspect that Pi-deficient plants will be more vulnerable than P-sufficient plants when they encounter additional stresses in their life span. In the future, it will be meaningful to investigate the potential of nutrient-stressed melons when enduring additional environmental constraints.

## Figures and Tables

**Table 1:** The number of starch grain, plastoglobuli, and grana stacks per chloroplast under the different Pi treatments. The values represent the mean ± SD (*n* > 30) and the asterisks indicate the significant differences according to Tukey’s test using a one-way ANOVA (* *p* < 0.05).

**Pi Supply/mM**	**Starch Grain**	**Plastoglobuli**	**Grana Stack**
0.25	0	4.64 ± 0.57	24.27 ± 2.53 *
0.001	2.41 ± 1.14 *	7.18 ± 0.43 *	16.01 ± 1.00

## Supplementary Materials

The following supporting information can be downloaded at: https://www.mdpi.com/article/10.3390/ijms232012073/s1.
